# A Machine Vision Rapid Method to Determine the Ripeness Degree of Olive Lots

**DOI:** 10.3390/s21092940

**Published:** 2021-04-22

**Authors:** Luciano Ortenzi, Simone Figorilli, Corrado Costa, Federico Pallottino, Simona Violino, Mauro Pagano, Giancarlo Imperi, Rossella Manganiello, Barbara Lanza, Francesca Antonucci

**Affiliations:** 1 Consiglio per la Ricerca in Agricoltura e L’Analisi Dell’Economia Agraria (CREA), Centro di Ricerca Ingegneria e Trasformazioni Agroalimentari, Via Della Pascolare 16, 00015 Monterotondo, Rome, Italy; luciano.ortenzi@crea.gov.it (L.O.); simone.figorilli@crea.gov.it (S.F.); corrado.costa@crea.gov.it (C.C.); federico.pallottino@crea.gov.it (F.P.); simona.violino@crea.gov.it (S.V.); mauro.pagano@crea.gov.it (M.P.); giancarlo.imperi@crea.gov.it (G.I.); rossella.manganiello@crea.gov.it (R.M.); 2 Consiglio per la Ricerca in Agricoltura e L’Analisi Dell’Economia Agraria (CREA), Centro di Ricerca Ingegneria e Trasformazioni Agroalimentari, Viale Lombardia C.da Bucceri, 65012 Cepagatti, Pescara, Italy; barbara.lanza@crea.gov.it

**Keywords:** k-NN, olive maturation index, image analysis, olive harvesting time, image color calibration

## Abstract

The degree of olive maturation is a very important factor to consider at harvest time, as it influences the organoleptic quality of the final product, for both oil and table use. The Jaén index, evaluated by measuring the average coloring of olive fruits (peel and pulp), is currently considered to be one of the most indicative methods to determine the olive ripening stage, but it is a slow assay and its results are not objective. The aim of this work is to identify the ripeness degree of olive lots through a real-time, repeatable, and objective machine vision method, which uses RGB image analysis based on a k-nearest neighbors classification algorithm. To overcome different lighting scenarios, pictures were subjected to an automatic colorimetric calibration method—an advanced 3D algorithm using known values. To check the performance of the automatic machine vision method, a comparison was made with two visual operator image evaluations. For 10 images, the number of black, green, and purple olives was also visually evaluated by these two operators. The accuracy of the method was 60%. The system could be easily implemented in a specific mobile app developed for the automatic assessment of olive ripeness directly in the field, for advanced georeferenced data analysis.

## 1. Introduction

The maturation stage of olives, both for oil and table uses, is one of the most important factors for organoleptic quality to be considered at harvest time [1,2]. As reported by Garcia et al. [3], olive ripening begins after a period of 25 weeks of cell growth, and during this time the fruit develops to its final size, maintaining the original green skin color. In this first stage (i.e., green stage), olives are most suitable for processing by any method as green table olives. The olives change from a leafy green, to a yellow-green, to a straw color, reaching their maximum size, ready for picking [4]. Following this, chlorophyll pigments are progressively replaced by anthocyanins (the turning color stage), and the process can be divided into different stages: “spotted stage”, “purple stage”, and a “black stage”, according to the content and to the skin color of the fruit [3,5,6]. Picked olives should be collected into 20–25 kg slotted trays, and picking the fruit at the correct ripeness stage (i.e., with a predetermined color degree) is the most important factor, assuring that only quality fruit is chosen [4]. As reported by Benalia et al. [7], not all the drupes of an orchard or tree turn color at the same time, so rapidly assessing the maturity index (MI) can help producers categorize their fruit’s maturity level. This allows evaluation of the varieties under specific growing conditions over several years, making the level of fruit maturity from year to year more easily replicable. The MI is calculated at the Venta del Llano Experimental Station, IFAPA, Mengíbar (Jaén index) [8] by randomly selecting 100 fruits out of a sample bucket and separating them into eight color categories based on a specific color chart from 0 to 7 (0 is equal to deep green skin color, 1 is equal to yellow-green, etc.). Subsequently, the number of fruits in each category is multiplied by the assigned number of the color category. Finally, all the numbers are added together and divided by 100:

(1) $$\mathrm{MI}=\frac{A\times 0+B\times 1+C\times 2+D\times 3+E\times 4+F\times 5+G\times 6+H\times 7}{100}$$

where the letters A–H are the number of fruits in each category. The index is indicative of the optimum maturation degree of specific cultivars.

As reported by Guzmán et al. [9], for small fruits such as olives, this kind of classification is based on the empirical visual evaluation of characteristics (e.g., skin color and defects presence), making this procedure very subjective and dependent on the experience of the evaluators, as well as on environmental factors. Quality raw olives are essential for good-quality oil and table olive production [10]. To reduce both the processing time and the subjectivity of the visual evaluation, automatic procedures were developed in the last decade to predict the ripening index of olive lots [11,12]. Machine vision classification and selection could be carried out automatically by fixing standard rules for grading criteria. This automatic sorting system could both save time and reduce manual labor [13].

The early detection and management of determined characteristics associated with the production line could increase yield and profit while decreasing human error. In addition, as reported by Smith [14], robotics and automated systems could remove much of the need for human decision-making and improve farm efficiencies. Generally, digital agriculture applications are suitable to evaluate systems on a holistic basis at multiple levels (e.g., individual, local, regional, and global), and generate tools that allow improved decision-making in every sub-process. Recently, several studies have been conducted in relation to automatic image analysis fruit classification. Neural networks are one of the most efficient approaches for the automatic identification of objects in images. Deep neural networks (DNNs) are characterized by many internal processing levels, combining the ability to recognize objects based on color, texture, and shape. There have been many studies on the use of convolutional neural networks, including fast and efficient algorithms of the You Only Look Once (YOLO) family. These methods unify target classification and localization into a regression problem, without requiring region proposal networks, and directly perform regression to detect targets from the image [15]. In the study of Kumar et al. [16], the whale optimization-based artificial neural network (WOANN) was used to predict distinct tomato diseases. The WOANN, prior to classifying diseases, performs color image segmentation through the firefly algorithm.

The aim of this work is to rapidly identify, immediately after harvesting, the ripeness degree of olive lots (both for olive oil and table olive purposes) placed in stackable plastic trays or on the ground (where these trays may be of several different capacities), to decide their destination in terms of the appropriate next steps in processing (de-bittering, etc.) through a potentially real-time, repeatable, and objective machine vision method. in this work, in order to overcome the different environmental situations that can occur in dynamic environments (olive oil mill, open air, etc.), pictures were subjected to an automatic colorimetric calibration method developed by Menesatti et al. [17]. This method consists of the management of simple RGB images calibrating their colors on the basis of the values of the 24 patches in the GretagMacbeth ColorChecker. A specific app for automatic olive ripeness calculation could be developed in future studies, to support the operation directly in the field, under different environmental situations that normally occur in dynamic environments, providing operators with an easy-to-use tool for smartphones. The speed of this method resides in the image being taken immediately after harvesting, with consecutive rapid potential evaluation returning the average ripeness degree for each olive tray.

## 2. Materials and Methods

### 2.1. Data Collection

The olive drupes (*Olea europaea*) of three cultivars (i.e., *Carboncella*, *Frantoio*, and *Leccino*) were collected in the olive groves of the “Ponzani Antonio” mill, located in Montorio Romano (Central Italy; N 42.1395917; E 12.77265), during the last olive harvest in November 2019.

The olives of the three cultivars were harvested in different stages of maturation following the peel colors. *Carboncella* and *Frantoio* were harvested at medium maturity, while *Leccino* was harvested at full maturity. While the collection of *Carboncella* was carried out (i) by hand, and (ii) facilitated using mechanical combs; for the other two cultivars the harvest was entirely mechanical. The olives were placed in 66 stackable plastic trays and then taken to the olive mill for the image acquisition. Of the 66 trays, 10 were visually evaluated by two operators (manual 1 and manual 2) for the identification of black, green, and purple olives. Table 1 reports the number of total trays (one image per tray) for the three cultivars, and the harvest typology (i.e., by hand or mechanical) used for the automatic and visual image analyses.

The harvested olives showed no infection and looked healthy. Generally, table olives are collected by hand to prevent damage to the olives due to mechanical harvesting [18], but this study was carried out keeping in mind both oil and table olives.

### 2.2. Image Processing

The olives placed in the 66 trays, divided for each cultivar, were taken to the olive mill and immediately a 5184 × 3888 pixel image was manually acquired for each tray, using a Sony Cyber-Shot DSCHX60 (CMOS, 20.4 Megapixel) digital camera, with no use of a steady support. The illumination was even, due to the pictures being taken inside of the oil mill with diffused light conditions. The acquisition scheme was quite simple and used just a normal tripod. Glare is optically present when shooting normally against light, and was easy to avoid in this case. No droplets or backlighting occurred. In addition, the light correction was done following a well-established algorithm, a procedure that was tested several times in the study of Menesatti et al. [17]. Before acquiring the images of the olive trays, on the first acquisition and at approximately every 5–6 subsequent acquisitions, a GretagMacbeth color checker (24 patches) was placed on the tray for automatic calibration of the image, in an effort to overcome the different environmental situations that can occur in the mill. Based on the a priori known patch values, all images were calibrated following the thin-plate spline interpolation function [19] in the red, green, blue (RGB) space and the procedure developed in MATLAB (rel 7.1; Mathworks, Natick, MA, USA) [17]. This procedure refers to a deflection in the z direction (orthogonal to the plane) of the transformation as a displacement of the x or y coordinates, in the plane in which the measured color checker sRGB coordinates of each image were warped (transformed), into the reference coordinates of the same color checker. This transformation was performed through a function modified for the three-dimensional space, the TPS interpolation. The three-dimensional sRGB color space is an additive color model in which red, green, and blue light are added together in various ways to reproduce a broad array of colors. A comparison with visual image evaluation was carried out by two selected operators (manual 1 and manual 2) in order to check the performance of the automatic machine vision method. This procedure was performed on ten trays and used as a validation set for the model.

**Figure 1:**
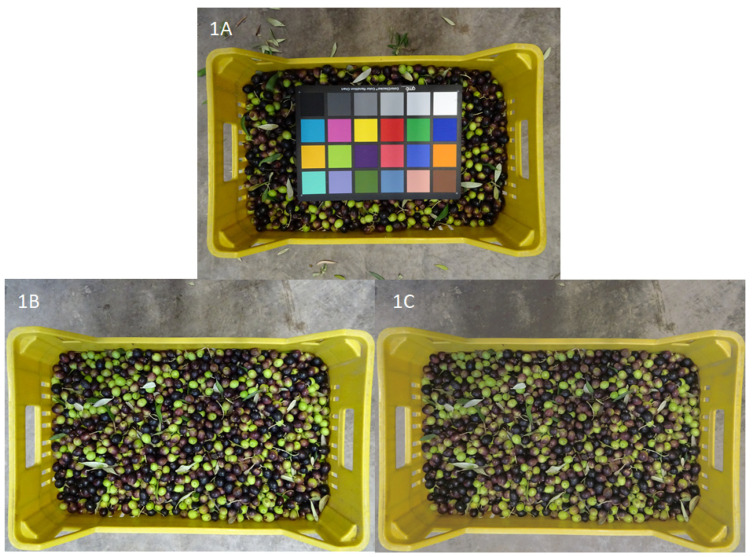
(**A**) Color checker GretagMacbeth (24 patches) within the olive tray; (**B**) original acquired image of olive tray; (**C**) resulting calibrated image of olive tray.

Figure 1 shows the color checker GretagMacbeth (24 patches) within the olive tray (Figure 1A), the comparison between the original acquired image (Figure 1B), and the resulting calibrated olive tray (Figure 1C).

### 2.3. Automatic Olive Ripeness Estimate

Once calibrated, the following pre-processing procedure was applied to each of the 66 images of the dataset: (1) transformation of the color coordinates from RGB into hue saturation value (HSV, which was found to be more efficient for the specific purpose after testing); (2) extraction of the H and S channels; (3) application of median filter. The result of this procedure applied to a single picture is a 5-channel RGBHS image. At this point, each of the 66 images collected were segmented using a k-nearest neighbors (k-NN) clustering algorithm for pixel classification [20]. The k-NN algorithm is a non-parametric classification method where the class of a given object is assigned based on the Euclidean distance (in an appropriate multidimensional space) with respect to k-neighbor objects from a subset for which the correct classification is known (training set) [21,22].

In this study, the training set was made of 1025 5-dimensional vectors and the number of neighbors *k* = 5. The 5-dimensional vectors were obtained by extracting the average color from 1025 patches (10 × 10 pixels each) processed with the procedure described above. The patches were extracted from the images (size 5184 × 3888 pixels) and are representative of the 7 classes. As a result, the training set was composed of 1025 patches (10 × 10 pixels per patch) in total, divided in groups: 138 for the background, 141 for the yellow tray, 121 for the red tray, 235 for the leaves, 138 for the black olives, 131 for the purple olives, and 121 for the green olives. The software utility (k-PE—k-NN Patches Extraction) was used to facilitate the patch extraction. The software acquires square patches of a chosen size and assigns them the class chosen by the user [20]. As the quality metric was selected, this meant that the accuracy (i.e., the ratio of correctly predicted observation to the total observations), the recall or sensitivity (i.e., the ratio of correctly predicted positive observations to all observations in actual class), and the precision (i.e., the ratio of correctly predicted positive observations to the total predicted positive observations) parameters were not considered.

## 3. Results

**Figure 2:**
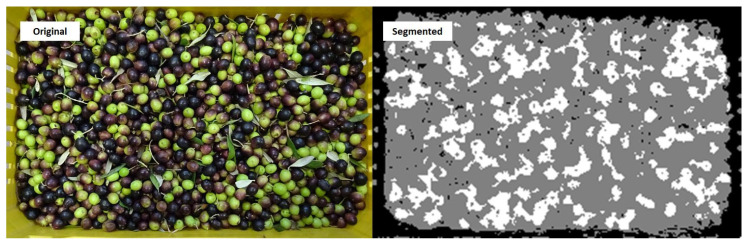
Comparison between the original image (**left**) and the segmented image (**right**) according to the 7-class k-nearest neighbors (k-NN) classification algorithm.

The classification of pixels allowed for the image segmentation shown in Figure 2, where white pixels correspond to ripe olives (black), gray pixels are associated with the unripe olives (green), and black pixels correspond to the other classes (red and yellow trays, leaves, background, and purple olives). Afterwards, the white pixels were counted, and the percentage of ripe olives was estimated by dividing the number of white pixels by the number of pixels associated with the black, green, and purple olives.

**Figure 3:**
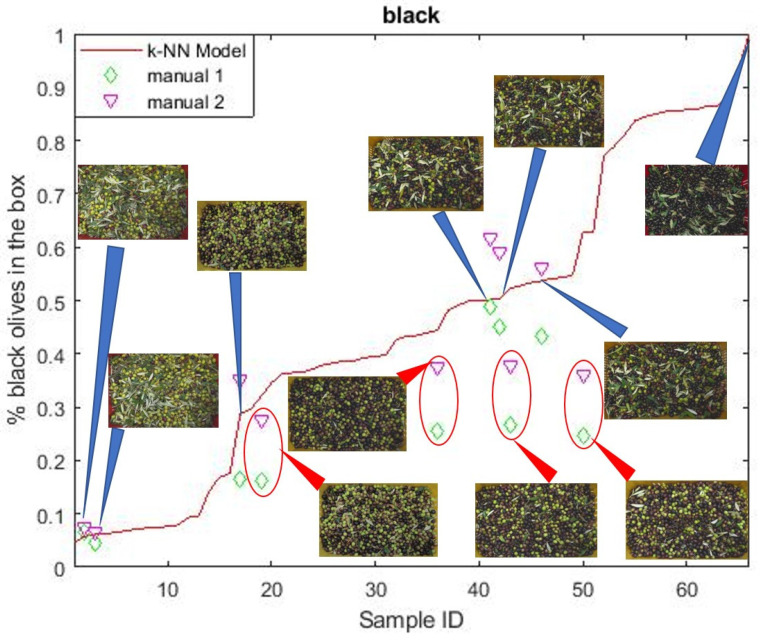
Comparison between the percentage of ripe (black) olives for each olive tray estimated by the counting algorithm (red line) and the percentage of ripe olives manually evaluated by two different operators: manual 1 (green diamonds) and manual 2 (purple triangles). The manual evaluation was performed on only 10 samples as a validation test of the counting algorithm. The percentage of black olives was overestimated for cleaner trays.

Figure 3 reports the estimated percentage of ripe (black) olives for each olive tray, sorted by increasing percentage of black olives. For 10 images, the number of black, green, and purple olives was also visually evaluated by two operators. These two sets are identified as manual 1 and manual 2 in Figure 3. The two sets are not in perfect agreement, and show a systematic trend: the percentages of the set identified as manual 1 are always smaller than those identified as manual 2. This is due to the different subjective perception of the maturation degree, typical of human operators. This intrinsic discrepancy defines a confidence interval for evaluating the algorithm, and the 10 samples manually evaluated were used as a validation set. The aim of the present work is to estimate the percentage of ripe olives in a tray from a picture of it. Therefore, at the present stage, the detection of a single ripe olive was not performed. As a result, the precision and recall quality metrics were not considered, whereas the accuracy in estimating the percentage of ripe olives in the tray was considered to be a performance indicator of the algorithm.

For six samples out of ten, the estimated percentage of black olives in the tray resided within the confidence interval. For the remaining four samples, the algorithm overestimated the percentage of black olives in the tray. As a result, the accuracy of the method was 60% in the validation.

This is probably due to the variability of the olive area aspect ratio across the samples. This problem could be easily overcome in future applications by means of a steady support for the camera. A further source of error comes from the misclassification of green olives, classified as “leaves”. When this happened, the percentage of green olives decreased in favor of the black olive percentage. As some trays had fewer leaves than others, the probability of green olives of being misclassified was not uniform across the samples. The four samples whose percentage of black olives was overestimated by the algorithm had fewer leaves (cleaner trays in Figure 3). This artefact can be easily corrected by cleaning the olive trays before classification, or at least making the percentage of leaves uniform across the different olive trays. This would ensure that the probability of misclassifying a green olive as a “leaf” is uniform for all trays, and the resulting systematic error could be easily eliminated by dividing for the maximum percentage.

## 4. Discussion

The influence of the harvesting date on olive quality and quantity, and the determination of the optimal harvest period in relation to yield and level of fruit maturation is crucial for growers. In addition, the ripening stage of olives can still represent some difficulties in terms of practical applicability and adaptability in different environments [23].

The Jaén index, evaluated according to the International Olive Oil Council method [8,24] by measuring the average coloring of olive fruits (peel and pulp), is currently considered one of the most indicative methods to determine the ripening stage of olives. As reported by Licausi et al. [25], using the optimal range of the Jaén index makes it possible to provide a direct approach (for different cultivars) to supply indications about the ripening phase of the fruit.

However, counting and evaluating the fruit pulp of 100 olives by eye is not a fast and objective method for real-time in-field ripeness assessment. Moreover, the index is indicative of the optimum degree of maturation of determinate cultivars.

For all these reasons, an image-analysis-based method was developed to extract meaningful information from digital images paired with advanced processing techniques [26]. This method allows large amounts of data to be processed and can solve tasks that require complex computation for the extraction of both quantitative and qualitative information [27]. In addition to these points, since the image analysis is based on instrumental measurements, it represents an objective and cheap non-destructive method to pursue the rapid evaluation of an entire stock of goods, resulting in the possibility of applying this method to a large number of samplings, increasing the repeatability and robustness of the measurements while overcoming the subjectivity of expert eye-based assessment.

In this study, the olive lot analyzed for ripeness degree definition (without considering the pulp as in the Jaén index) was directly photographed at harvest time, after positioning the olives in stackable plastic trays. It is at this part of the harvesting process when the potentially rapid real-time machine vision method could be applied in practice, once implemented. Furthermore, the analytical technologies used were made objective and repeatable thanks to the automatic colorimetric calibration method, which consists of the management of simple RGB images by calibrating their colors on the basis of the 24 patch values of the GretagMacbeth ColorChecker. The original images were processed using pixel values classified by thresholding and a segmentation algorithm, as an artificial vision technique for the automatic detection of the external quality of olive fruits [28]. In addition, even if the accuracy is 60%, the automatic method developed in this study could compensate for both human eye error and objectivity, as the definition of the ripeness degree of olive lots is currently assessed only by eye, as per the Jaén index.

The organoleptic quality of olives (both for oil and table) is influenced by several factors: geographical production area, climatic conditions, cultivar, and harvesting time [29]. In particular, the effects of harvesting time are of particular interest to the grower because the improper selection of harvest time could compromise the final product quality [3,30]. As reported by Dag et al. [23], the growth of intensive orchard practices and the development of rapid tools for mechanized harvesting have brought about the need to rapidly and objectively determine the fruit maturity in relation to cultivar, environmental conditions, and agronomic practices. This is reflected in the increasing demand for rapid, real-time, and objective methods for determining the ripeness of entire olive lots directly in field after harvesting. In fact, non-destructive technologies for the early assessment of fruit ripeness on the plant, and the prediction of the harvest dates, have been of growing interest, with the potential to revolutionize agricultural practices since they provide numerous advantages over traditional destructive methods [31].

In this context, recent improvements in machine vision systems have provided new possibilities for applications in agriculture. For example, in the study of Wan et al. [32], the maturity level detection of fresh tomatoes, by combining the feature color values with the back-propagation neural network (BPNN) classification technique has been developed. Computer vision systems can also be used as a tool to select tomatoes by detecting defects through a radial basis function-support vector machine (RBF-SVM) classifier that uses the pixel values of the LAB color space, creating a relationship between the image and the defect [33]. Kuznetsova et al. [34] developed a computer vision system based on a YOLOv3 algorithm with special pre- and post-processing to effectively detect apples in orchards in a very short time using harvesting robots. In their study, YOLOv3 with pre- and post-processing was able to detect 90.2% of apples in orchards (recall = 90.2%), with the objects mistaken for apples of 7.8% (precision = 92.2%).

The proposed method based on simple RGB images and on a k-NN clustering algorithm could be a valid tool to overcome the slowness and subjectivity of the approaches entirely based on the visual counting of the olive maturation index by manual operators. In addition, this method could overcome the human perception that has difficulty in correctly estimating small and dark objects, which could affect the final objective estimation. Finally, this automated method could compensate for human eye error and objectivity, and rapidly simplify the assessment of processing destination of freshly harvested olive lots, by taking photos directly in the olive oil mill.

## 5. Conclusions

Being that this a preliminary study, it is a first approach to simplify the processing destination of freshly harvested olives lots for farmers. A specific app (DEAOLIVA-INNOLIVAPP) for the automatic olive ripeness calculation will be developed, providing operators with a simple and easy-to-use tool for smartphones, once the procedure is validated on a larger dataset over a period of at least three years, to support the operation directly in field (mill, open air, etc.) with different environmental situations that can occur in dynamic environments. Performing the test with additional samples in the future will be necessary to test more accurate machine learning algorithms. Even if the accuracy is only 60%, this parameter was chosen as the only quality metric of the algorithm rather than recall and precision, as it is more intuitive for an immediate response on the app that will be developed in the future. The information registered through the app, will be stored on an internal database and subsequently synchronized on a remote server. Internet connectivity will be needed for the synchronization on the remote server, which could be achieved both in field (if Internet connection is available) or afterwards, when field operations are concluded and Internet signal becomes available. These methods, being highly objective, could also be integrated to certify the olive ripeness through blockchains, for example, to guarantee the quality of the product to the consumer. In addition, in light of the recent developments in precision farming and digital agriculture applications, the specifically developed mobile app could include advanced features; for example, in order to correlate the ripeness of the fruit, georeferenced data with meaningful farming data could be used to adjust and optimize agronomical practices, or for planning a differentiated harvesting strategy in the future on the basis of time-series data. This would lead to a more efficient farming system and higher-quality production.

## Figures and Tables

**Table 1:** Number of images/trays and harvest typology (i.e., by hand or mechanical) for each cultivar (i.e., *Frantoio*, *Carboncella*, and *Leccino*) automatically and visually analyzed in the present study.

	CV	N Images/Trays	Harvest	
			by Hand	Mechanical
Automatic evaluation	*Frantoio*	13	0	13
	*Carboncella*	51	35	16
	*Leccino*	2	0	2
	TOT	66	35	31
Visual evaluation	*Frantoio*	2	0	2
	*Carboncella*	8	8	0
	*Leccino*	0	0	0
	TOT	10	8	2
